# GLP-1 receptor agonists and psychiatric outcomes: a bibliometric analysis of research trends, thematic evolution, and collaboration networks

**DOI:** 10.3389/fpsyt.2026.1857078

**Published:** 2026-07-21

**Authors:** Maria Meritxell Roca Mora, Marcia Sanchez-Monge, Dibe Balardini Ayoub, Ana Beatriz P. Aguiar-Barros, Sofia Serrano-Porras, Renato de Filippis, Georgios Schoretsanitis

**Affiliations:** 1 Fundación Hospitalarias, Sant Boi de Llobregat, Barcelona, Spain; 2 Parc Sanitari Sant Joan de Déu, Sant Boi de Llobregat, Barcelona, Spain; 3 School of Medicine, Universidad Peruana Cayetano Heredia, Lima, Peru; 4 Department of Medicine, Universidade do Extremo Sul Catarinense, Criciuma, Brazil; 5 Department of Medicine, Cesmac University Center, Maceio, Brazil; 6 Faculty of Health Sciences, Universidad Internacional de la Rioja, Logroño, Spain; 7 Department of Health Sciences, University “Magna Graecia” of Catanzaro, Catanzaro, Italy; 8 Department of Psychiatry, Unit of Pharmacogenetics and Clinical Psychopharmacology, Centre for Psychiatric Neuroscience, Lausanne University Hospital, University of Lausanne, Lausanne, Switzerland; 9 Psychiatric University Hospital Zurich, Department of Psychiatry, Psychotherapy and Psychosomatics, University of Zurich, Zurich, Switzerland; 10 Department of Psychiatry, The Zucker Hillside Hospital, Behavioral Health Pavilion, Northwell Health, Glen Oaks, NY, United States; 11 Department of Psychiatry, Zucker School of Medicine at Northwell/Hofstra, Hempstead, NY, United States

**Keywords:** bibliometric analysis, depression, GLP-1 receptor agonists, psychiatry, science mapping, semaglutide, substance use disorders

## Abstract

**Introduction:**

Research on glucagon-like peptide-1 receptor agonists (GLP-1 RAs) and psychiatric outcomes has expanded rapidly following the clinical success of semaglutide, pharmacoepidemiological signals on psychiatric adverse events, and growing regulatory scrutiny, yet the intellectual structure and thematic evolution of this interdisciplinary field remain uncharacterised. We aimed to map publication trends, conceptual structure, thematic evolution, and collaboration networks of research on GLP-1 RAs and psychiatric outcomes.

**Methods:**

Bibliographic records were retrieved independently from OpenAlex, Lens.org, and PubMed (MEDLINE) on 8 April 2026, using parallel Boolean searches restricted to title and abstract fields. After cross-database deduplication and cleaning, 2, 197 documents (1990--2026) were retained. Performance analysis, science mapping (keyword co-occurrence, collaboration networks), and thematic analysis (Callon’s centrality-density map, thematic evolution) were performed using the bibliometrix R package.

**Results:**

The field showed exponential growth, with annual output rising from fewer than 10 publications before 2004 to nearly 500 in 2025. The corpus spanned 1, 063 journals, 9, 269 authors, and 85 countries (h-index 101; mean 21.9 citations/document). Thematic mapping placed depression in the niche themes quadrant (high density, low centrality), while obesity and diabetes mellitus occupied the basic themes quadrant. Thematic evolution showed depression emerging as a distinct research front only in 2022--2026.

**Discussion:**

Depression research within the GLP-1 RA literature has developed internal coherence as a niche theme but remains peripheral to the dominant metabolic research front. Bridging this gap through large cardiometabolic trials with psychiatric co-primary endpoints represents a key priority for future research.

## 1 Introduction

Glucagon-like peptide-1 receptor agonists (GLP-1 RAs) are incretin-based therapies originally developed for type 2 diabetes mellitus and, more recently, obesity (1, 2). Agents such as semaglutide, liraglutide, dulaglutide, and exenatide enhance glucose-dependent insulin secretion, suppress glucagon release, and promote satiety through central appetite regulation (3). Beyond their metabolic indications, accumulating evidence has linked GLP-1 RA use to effects on psychiatric outcomes including depression, anxiety, substance use disorders (SUDs), eating disorders, suicidality, and cognitive function (4). GLP-1 receptors are expressed throughout the central nervous system, including the mesolimbic reward pathway, hippocampus, and prefrontal cortex, providing a plausible neurobiological basis for these effects (5, 6). Recent large-scale studies have supported these observations: Xie et al. (7) reported reduced risks of SUDs, psychotic disorders, and neurocognitive disorders among 215, 970 GLP-1 RA users; a Swedish national cohort study found a 42% reduced risk of worsening psychiatric outcomes with semaglutide specifically in individuals with depression or anxiety (32); and a meta-analysis of 80 randomised controlled trials characterised the psychiatric adverse event profile of these agents (4).

As this literature has grown rapidly, narrative and systematic reviews have provided useful syntheses of clinical evidence (4, 8), but these are designed to evaluate treatment effects rather than characterise the research landscape. Bibliometric analysis offers a complementary approach by quantifying publication trends, identifying intellectual and social structures, and mapping collaboration networks across an entire field (9, 10). Through techniques such as keyword co-occurrence analysis, thematic mapping, and co-authorship network analysis, bibliometric methods can reveal emerging research frontiers and structural gaps that are not visible from individual reviews. Unlike a conventional systematic review, the present study synthesises the characteristics of the literature itself rather than the clinical effects reported within it.

Previous bibliometric analyses of GLP-1 RA research have focused on semaglutide broadly (11), cardiovascular disease (12, 13), obesity management (14), and tirzepatide pharmacology (15). None has focused on psychiatric or mental health outcomes, a notable gap given the rapidly growing clinical and regulatory interest in the neuropsychiatric effects of this drug class and the interdisciplinary nature of the field, which spans endocrinology, psychiatry, neuroscience, pharmacology, and addiction medicine.

We aimed to perform the first bibliometric analysis of GLP-1 RAs and psychiatric outcomes, specifically to: (1) characterise publication trends and growth patterns from 1990 to 2026; (2) identify leading contributors; (3) map the conceptual structure through keyword co-occurrence and thematic analysis; (4) trace thematic evolution across three time periods; and (5) delineate collaboration networks at the country and institutional levels.

## 2 Methods

### 2.1 Study design and reporting framework

We conducted a bibliometric analysis following the methodological framework proposed by Donthu et al. (9) for conducting bibliometric research in business and the social sciences, adapted here for biomedical literature. Although this article is classified as a systematic review, it is a bibliometric mapping study rather than a conventional evidence-synthesis systematic review: it systematically characterises the volume, structure, and evolution of the published literature rather than pooling or critically appraising the treatment effects reported within individual studies. Accordingly, we followed a systematic and reproducible search and screening procedure but did not perform quantitative outcome synthesis, risk-of-bias assessment, or effect-size estimation. No protocol was registered for this bibliometric analysis, as bibliometric analyses are not eligible for PROSPERO registration. The search strategy and analytical pipeline were finalized prior to data collection and are publicly available. The analysis incorporated three complementary approaches: performance analysis (descriptive metrics of research output), science mapping (network-based analysis of intellectual and social structures), and thematic analysis (conceptual mapping of research topics and their evolution). Analyses were conducted using the bibliometrix R package version 5.2.1 (10) in R version 4.5.2 (16).

### 2.2 Database selection and justification

To ensure broad coverage and minimise single-source bias, we retrieved records independently from three complementary bibliographic databases: OpenAlex (17), Lens.org, and PubMed (MEDLINE). Each was selected for distinct analytical strengths. OpenAlex is a fully open scholarly database indexing over 200 million works, with biomedical metadata coverage validated as comparable to proprietary databases (18, 19), and supports programmatic reproducible retrieval via a documented application programming interface (API) through the openalexR R package version 3.0.1 (20). Lens.org aggregates PubMed, Crossref, Microsoft Academic, and additional sources into a multidisciplinary index with structured Boolean field-level search. PubMed (MEDLINE) provides authoritative, curator-indexed biomedical coverage with MeSH-based subject tagging and deep historical depth in psychiatric and pharmacological journals. Together, these three databases offer a comprehensive, fully open, and reproducible evidence base for mapping the intersection of GLP-1 receptor agonists and psychiatric outcomes, a methodological choice that itself represents an advance over the single-database bibliometric analyses that dominate the field.

### 2.3 Search strategy

Searches were executed on 26 March 2026 (OpenAlex) and 8 April 2026 (Lens.org and PubMed). All three searches applied a two-block Boolean strategy combining GLP-1 receptor agonist drug terms (Block 1: GLP-1, GLP1, GLP-1RA, glucagon-like peptide-1, semaglutide, liraglutide, dulaglutide, exenatide, tirzepatide, lixisenatide, incretin) with psychiatric outcome terms (Block 2: 35 terms spanning psychiatric disorders, mood disorders, anxiety, suicidality, eating disorders, and substance use disorders; full list and the exact per-database query syntax provided in **Supplementary Materials (Section S0)**). Searches were restricted to title and abstract fields to ensure comparability across databases. The OpenAlex search used the title_and_abstract.search filter with manually expanded morphological variants, as OpenAlex does not support wildcard truncation; Lens.org and PubMed used wildcard truncation (e.g., psychiatr*, depress*), and PubMed applied [tiab] field tags. Lens.org output was filtered *post-hoc* in R to records with both drug and psychiatric terms present in title or abstract, matching the scope of the other two databases. No language restriction was applied, although English search terms introduced an inherent bias toward English-language literature.

As a worked example, the complete OpenAlex query combined the two blocks as two logically ANDed title_and_abstract.search filters:

filter=title_and_abstract.search:GLP-1|GLP1|GLP-1RA|glucagon-like peptide-1|glucagon-like peptide 1|semaglutide|liraglutide|dulaglutide|exenatide|tirzepatide|lixisenatide|incretin, title_and_abstract.search:psychiatric|psychiatry|neuropsychiatric|mental health|mental illness|depression|depressive|antidepressant|anxiety|suicidal|suicide|bipolar|mania|psychosis|psychotic|schizophrenia|antipsychotic|mood disorder|eating disorder|anorexia|bulimia|binge eating|addiction|substance use disorder|alcohol use disorder|opioid use disorder|smoking cessation|gambling disorder

The complete syntax for all three databases, including the full 35-term psychiatric block, is provided in **Supplementary Materials (Section S0)**.

### 2.4 Eligibility criteria

Records were included if they contained at least one term from each search block in the title or abstract and were published between 1990 and March 26, 2026. The year 1990 was selected as the lower bound because GLP-1 was characterized in the late 1980s, with the earliest relevant publications appearing in the early 1990s. Eligible document types included articles, reviews, letters, editorials, book chapters, and preprints. These types were retained deliberately, because bibliometric mapping aims to describe the full body of published work in a field, including the editorials, commentaries, and letters that often carry early regulatory debate and emerging clinical signals, rather than to synthesise treatment effects from primary studies alone. To ensure that this inclusive scope did not bias the results, we conducted a sensitivity analysis restricted to original articles and reviews (Section 2.7). Excluded document types were errata, data papers, paratext, dissertations, peer-review reports, and datasets.

### 2.5 Data cleaning and deduplication

Author names were harmonised across sources to a common “SURNAME INITIALS” format prior to merging via the mergeDbSources function in bibliometrix, which performs cross-database deduplication by DOI first and then by normalised title. A systematic cleaning pipeline was then applied to the merged dataset: removal of retracted publications; exclusion of records missing titles or publication years or published before 1990; normalisation of document type labels and filtering to eligible types (Article, Review, Editorial, Letter, Book Chapter, Preprint); removal of records from noisy or non-journal sources (preprint servers and indexing artifacts); additional deduplication using normalised DOIs and normalised (lowercased, alphanumeric-only) titles; and removal of records with no identified authors.

### 2.6 Data conversion and keyword processing

OpenAlex records were converted to the bibliometrix data format using a custom R script (21); Lens CSV exports were imported via convert2df(dbsource = “lens”) and PubMed.nbib files via convert2df(dbsource = “pubmed”). OpenAlex does not provide traditional author-supplied keywords, so machine-assigned keywords with relevance score ≥0.4 were used as a proxy. Lens and PubMed provide author-supplied keywords and MeSH descriptors respectively. A unified generic-term blacklist was applied post-merge to remove field-of-study terms (e.g., “medicine”, “biology”), methodological MeSH headers (e.g., “humans”, “male”, “cohort studies”, “placebo”), and other non-substantive terms.

### 2.7 Performance analysis

Descriptive performance metrics were computed using the biblioAnalysis function in bibliometrix. These included: total documents, annual publication counts and growth rates, document type distribution, most productive authors, most productive journals, most productive countries (based on author affiliation), average and median citations per document, the h-index of the field, the collaboration index (mean number of authors per paper), and the percentage of international co-authorships. To confirm that the inclusion of non-primary document types (editorials, letters, book chapters, and preprints; 11.4% of the corpus) did not distort the findings, we repeated the descriptive metrics and the top keyword ranking on the subset restricted to original articles and reviews (n = 1, 947) and compared them with the full corpus (**Supplementary Tables 4**, **5**). Bradford’s Law of scattering (22) was applied to identify the core journals of the field. Lotka’s Law of author productivity (23) was assessed by fitting the inverse generalized power law to the author frequency distribution, with goodness-of-fit evaluated using the Kolmogorov-Smirnov test.

### 2.8 Science mapping

#### 2.8.1 Keyword co-occurrence analysis

A keyword co-occurrence network was constructed from the filtered keywords (Section 2.6), displaying the top 20 keywords by frequency. Communities were identified using the Louvain algorithm (24); for visual clarity, edges with a co-occurrence weight below 5 were suppressed. Visualization was performed using the Fruchterman-Reingold layout.

#### 2.8.2 Country collaboration map

Country-level co-authorship was derived from author-affiliation country codes and visualised as a world map: countries were positioned at their geographic centroids, node size scaled to each country’s total collaboration links, and arc thickness set proportional to the number of co-authored documents between country pairs; the 45 strongest country pairs (minimum two co-authored documents) were displayed.

#### 2.8.3 Institution collaboration network

A co-authorship network at the institutional level was constructed from author affiliations, displaying the top 15 institutions by document count (a tighter cap than the country analysis, reflecting the greater density of institutional co-authorship). Clustering was performed using the Louvain algorithm.

#### 2.8.4 Three-fields plot

An alluvial diagram linking the top 10 authors, top 10 keywords, and top 10 journals was generated to visualize the relationships between key contributors, thematic areas, and publication venues.

### 2.9 Thematic analysis

#### 2.9.1 Thematic map

A strategic diagram (Callon’s centrality-density map) (25) was generated using the thematicMap function in bibliometrix, with keyword data (DE field), a maximum of 250 keywords, a minimum frequency threshold of 5, and no stemming. Themes were classified into four quadrants: motor themes (high centrality, high density), basic themes (high centrality, low density), niche themes (low centrality, high density), and emerging or declining themes (low centrality, low density).

#### 2.9.2 Thematic evolution

The evolution of research themes over time was analyzed using the thematicEvolution function, with three time periods defined by field milestones: 1990--2015 (preclinical and early pharmacological era), 2016--2021 (pharmacovigilance and translational era), and 2022--2026 (clinical evidence explosion). The top 100 keywords with a minimum frequency of 3 were included. Results were visualized as an alluvial diagram.

### 2.10 Software

**Figure 1:**
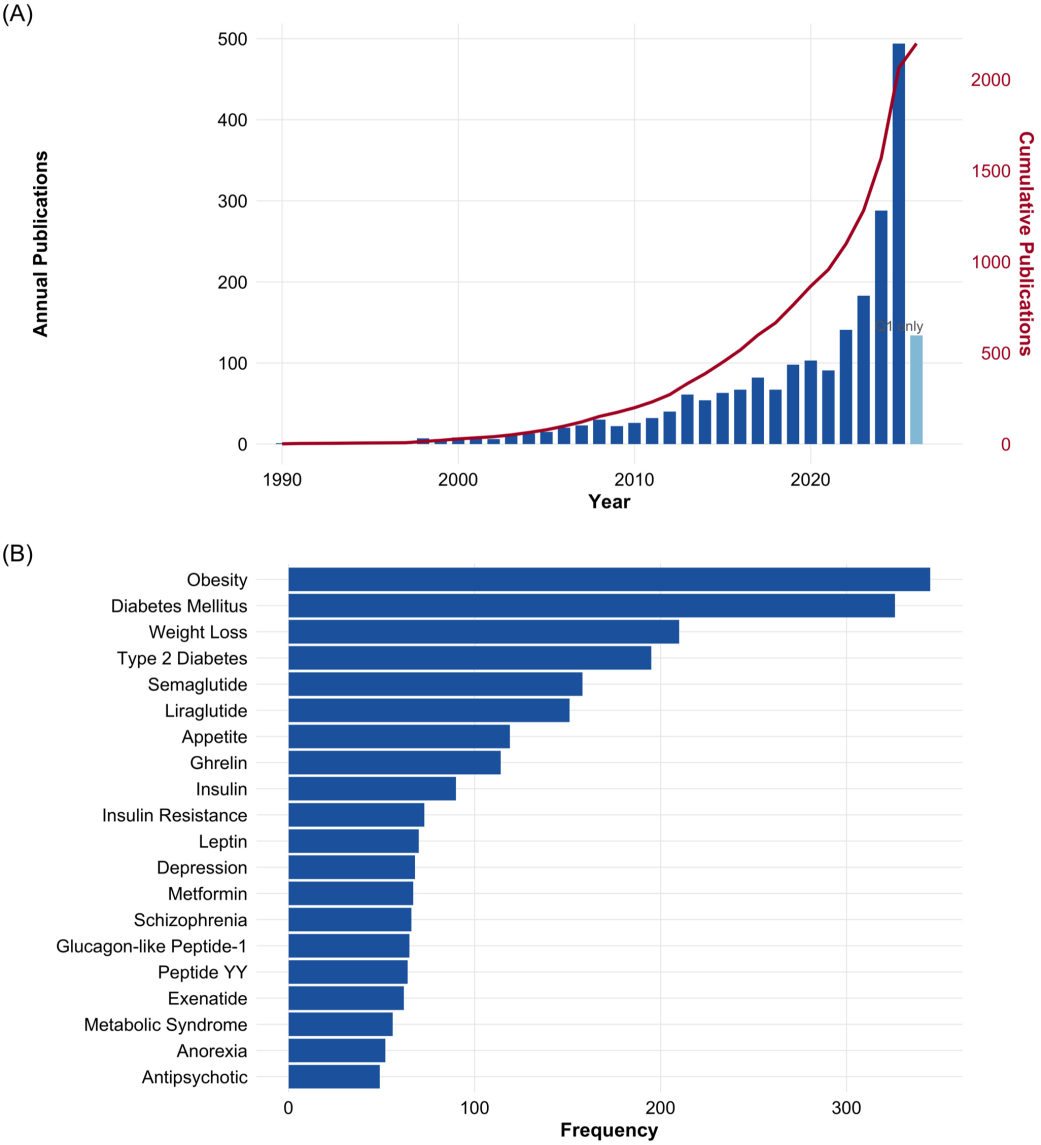
Research output and keyword profile. **(A)** Annual scientific production (1990--2026). Blue bars show annual document count (left axis), with 2026 in light blue (Q1 only). The red line shows cumulative production (right axis). **(B)** Top 20 machine-assigned keywords by frequency (filtered to relevance score of 0.4 or greater, generic terms removed).

**Figure 2:**
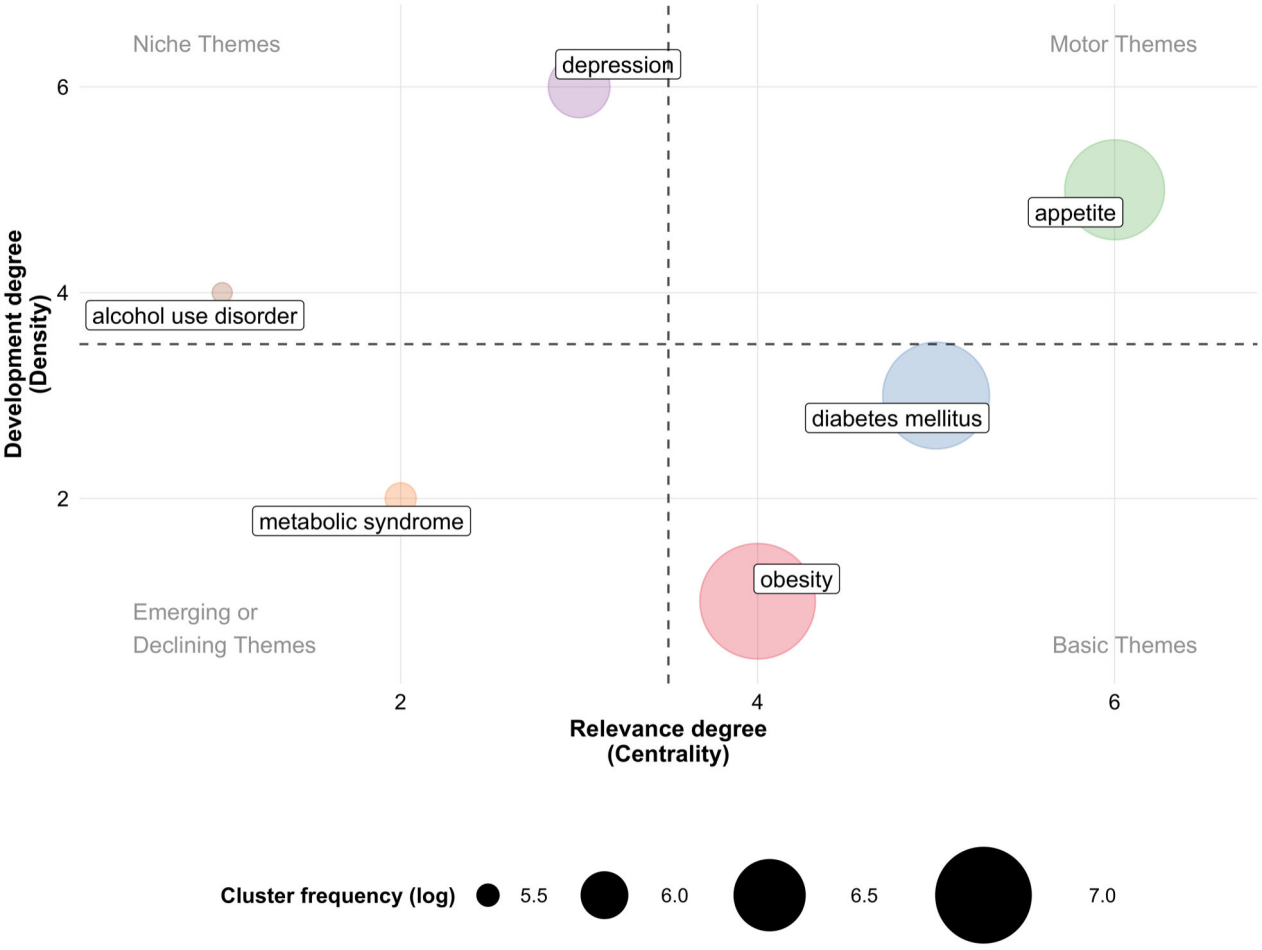
Thematic map (Callon’s centrality-density strategic diagram). Each bubble represents a keyword cluster. The x-axis (centrality) indicates the strength of the cluster’s connections to other themes; the y-axis (density) indicates the internal coherence of the cluster. Motor themes (upper right) are both important and well-developed. Basic themes (lower right) are important but underdeveloped. Niche themes (upper left) are well-developed but peripheral. Emerging or declining themes (lower left) are neither central nor cohesive.

All analyses were performed in R version 4.5.2 (16) using the bibliometrix package version 5.2.1 (10) and the openalexR package version 3.0.1 (20) for data retrieval. The keyword and institution co-occurrence networks (**Figure 1**, **Supplementary Figure 4**) were generated with the ggraph, ggrepel, and igraph packages, using Louvain community detection. The country collaboration map (**Supplementary Figure 2**) was rendered with ggplot2 and the maps package. The thematic evolution and three-fields alluvial diagrams (**Figure 2**, **Supplementary Figure 5**) were rendered with the ggalluvial package. The R code for this analysis is available at https://github.com/meritxellrocamora/glp1-psychiatry-bibliometric.

### 2.11 Ethics and patient and public involvement

#### 2.11.1 Ethics statement

This study is a bibliometric analysis of publicly available bibliographic metadata and does not involve human participants, human tissue, identifiable personal data, or animal research. Ethical approval was therefore not required.

#### 2.11.2 Patient and public involvement

Patients and the public were not involved in the design, conduct, reporting, or dissemination of this bibliometric analysis, as it is a secondary analysis of publicly available bibliographic metadata.

## 3 Results

### 3.1 Data retrieval and screening

**Figure 3:**
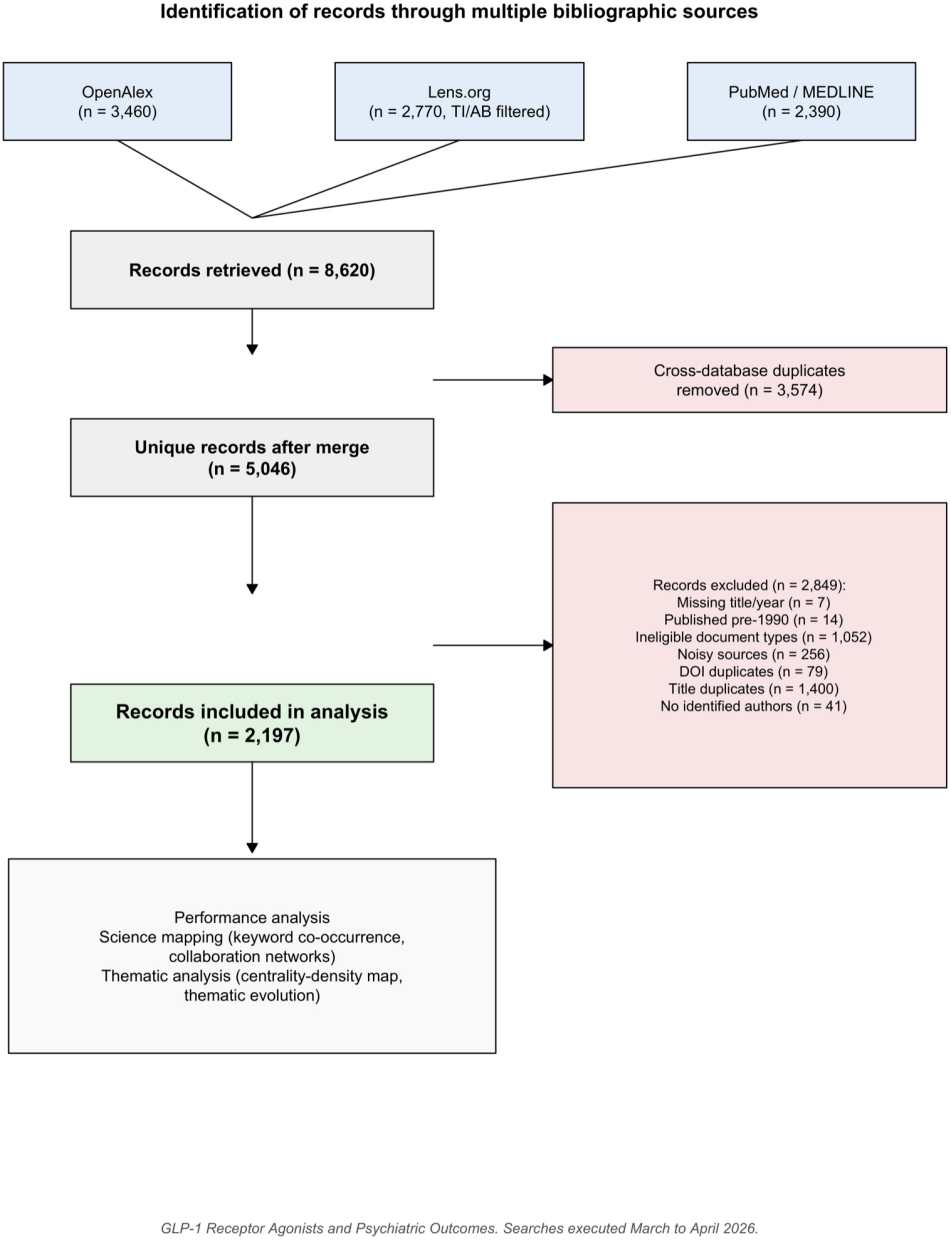
PRISMA-style flow diagram for data retrieval and cleaning. Records retrieved from OpenAlex (n = 3, 460), Lens.org (n = 2, 770 after *post-hoc* filtering to title/abstract matches), and PubMed (n = 2, 390), totaling 8, 620 records pre-merge. Cross-database deduplication removed 3, 574 duplicates, yielding 5, 046 unique records. Subsequent cleaning excluded: missing title/year (n = 7), pre-1990 (n = 14), ineligible document types (n = 1, 052), noisy sources (n = 256), additional DOI duplicates (n = 79), additional title duplicates (n = 1, 400), and no-author records (n = 41). Records included in analysis: n = 2, 197.

The three parallel searches retrieved 3, 460 records from OpenAlex, 2, 770 records from Lens.org (after *post-hoc* filtering to title/abstract matches), and 2, 390 records from PubMed (8, 620 records total pre-merge). Cross-database deduplication by DOI and normalized title via mergeDbSources removed 3, 574 duplicates, yielding 5, 046 unique records. Subsequent cleaning excluded 2, 849 additional records: 7 with missing title or publication year, 14 published before 1990, 1, 052 of ineligible document types, 256 from noisy or non-journal sources, 79 additional DOI duplicates, 1, 400 additional normalized-title duplicates, and 41 records with no identified authors. The final dataset comprised 2, 197 documents included in the analysis (**Figure 3**).

### 3.2 Descriptive overview

The 2, 197 documents were published between 1990 and 2026 across 1, 063 journals by 9, 269 authors affiliated with institutions in 85 countries (**Table 1**). The corpus consisted of 1, 658 articles (75.5%), 289 reviews (13.2%), 106 letters (4.8%), 105 editorials (4.8%), 36 book chapters (1.6%), and 3 preprints (0.1%). The field-level h-index was 101. The average number of citations per document was 21.9 (median: 1). The collaboration index was 5.24 authors per paper, and 14.1% of documents involved international co-authorship. A sensitivity analysis restricting the corpus to original articles and reviews (n = 1, 947; 88.6%) reproduced the overall structure of these findings: the timespan, the field-level h-index (100 versus 101), and the ten most frequent keywords were essentially unchanged, the two keyword rankings differing only in the ordering of the second- and third-ranked terms (**Supplementary Tables 4**, **5**).

**Table 1:** Main information of the dataset.

Metric	Value
Timespan	1990--2026
Databases	OpenAlex, Lens.org, PubMed
Documents	2, 197
Articles	1, 658
Reviews	289
Letters	106
Editorials	105
Book chapters	36
Preprints	3
Sources (journals)	1, 063
Authors	9, 269
Countries	85
Average citations per document	21.9
Median citations per document	1
h-index (field level)	101
Collaboration index (authors/paper)	5.24
International co-authorship	14.1%

### 3.3 Annual publication trends

The field demonstrated a pattern of exponential growth (**Figure 1A**). Annual output remained below 10 publications per year from 1990 to 2003. A steady growth phase followed, with 20 to 60 publications per year from 2004 to 2015. Output accelerated from 2016 to 2022 (60–150 publications per year), then underwent explosive expansion from 2023 onward, reaching nearly 500 publications in 2025. In the first quarter of 2026 alone, over 130 documents had already been indexed.

### 3.4 Most cited documents

The five most globally cited documents in the corpus were: Desborough (2000) on the stress response to trauma and surgery (TC = 2, 221), Rinella et al. (2023) on NAFLD practice guidance (TC = 2, 040), Garvey et al. (2016) on clinical practice guidelines for obesity (TC = 1, 755), Musso et al. (2010) on obesity, diabetes, and gut microbiota (TC = 662), and Asarian & Geary (2013) on sex differences in the physiology of eating (TC = 505). Citation counts reflect global citations at the time of data extraction.

### 3.5 Leading contributors

Bradford’s Law analysis identified 62 core journals in Zone 1 across the 1, 063 journals in the corpus. The top sources spanned endocrinology, metabolism, psychiatry, neuroscience, and pharmacology venues (**Supplementary Table 1**, **Supplementary Figure 1A**).

The top-ranked contributing countries are shown in **Supplementary Table 2** and **Supplementary Figure 1B**. The most prolific author was McIntyre R S (n = 16), a clinical psychiatrist whose work focuses on mood disorders and metabolic psychiatry, followed by Fink-Jensen A (antipsychotic-related metabolic effects) and Jerlhag E (GLP-1 and addiction neuroscience) (**Supplementary Table 3**). Author counts were computed after cross-database name harmonisation to a common “SURNAME INITIALS” format to ensure consistency across source databases.

### 3.6 Keyword analysis

The 20 most frequent machine-assigned keywords (filtered to relevance score of 0.4 or greater, generic terms excluded) were led by diabetes mellitus (n = 592), type 2 diabetes (n = 425), obesity (n = 405), and weight loss (n = 288) (**Figure 1B**). Psychiatric-specific keywords included depression (n = 111), addiction (n = 98), schizophrenia (n = 94), and alcohol use disorder (n = 88), with 5 of the top 20 keywords (25%) directly related to psychiatric outcomes.

### 3.7 Keyword co-occurrence network

**Figure 4:**
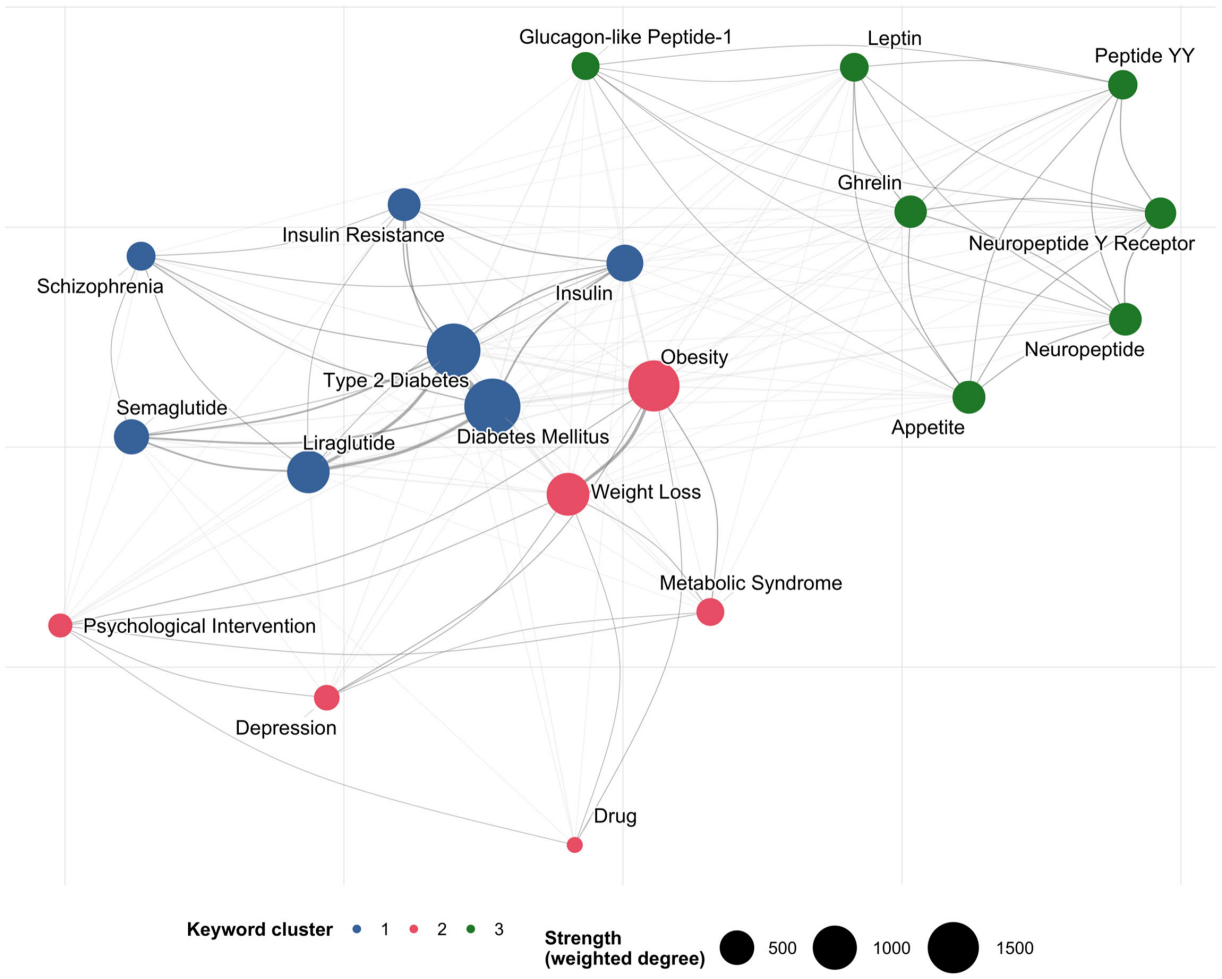
Keyword co-occurrence network. The top 20 keywords are displayed, with edges representing co-occurrence on the same document. Node size is proportional to keyword strength (weighted degree). Colors represent communities identified by the Louvain algorithm; edges with a co-occurrence weight below 5 are suppressed for legibility.

Keyword co-occurrence analysis identified three major thematic clusters (**Figure 4**). The first cluster (GLP-1 RA pharmacology/diabetes) encompassed diabetes mellitus, type 2 diabetes, liraglutide, semaglutide, exenatide, and glucagon-like peptide-1. The second cluster (clinical/psychiatric) included depression, obesity, weight loss, schizophrenia, anxiety, appetite, ghrelin, neuropeptide, and psychological intervention. The third cluster (metabolic mechanisms) comprised insulin, insulin resistance, metabolic syndrome, and type 2 diabetes mellitus. Addiction-related keywords (drug, alcohol use disorder) were distributed across the pharmacological and clinical clusters rather than forming a separate community.

### 3.8 Thematic map

The strategic diagram (Callon’s centrality-density map) classified the field’s research themes into four quadrants (**Figure 2**). The appetite cluster occupied the motor themes quadrant (high centrality, high density), reflecting its central and well-developed position in the field. The depression cluster occupied the niche themes quadrant (high density, low centrality), indicating a well-developed but relatively specialized research community. Two clusters occupied the basic themes quadrant (high centrality, low density): obesity and diabetes mellitus. Metabolic syndrome and alcohol use disorder clusters appeared in the emerging or declining themes quadrant (low centrality, low density).

### 3.9 Thematic evolution

**Figure 5:**
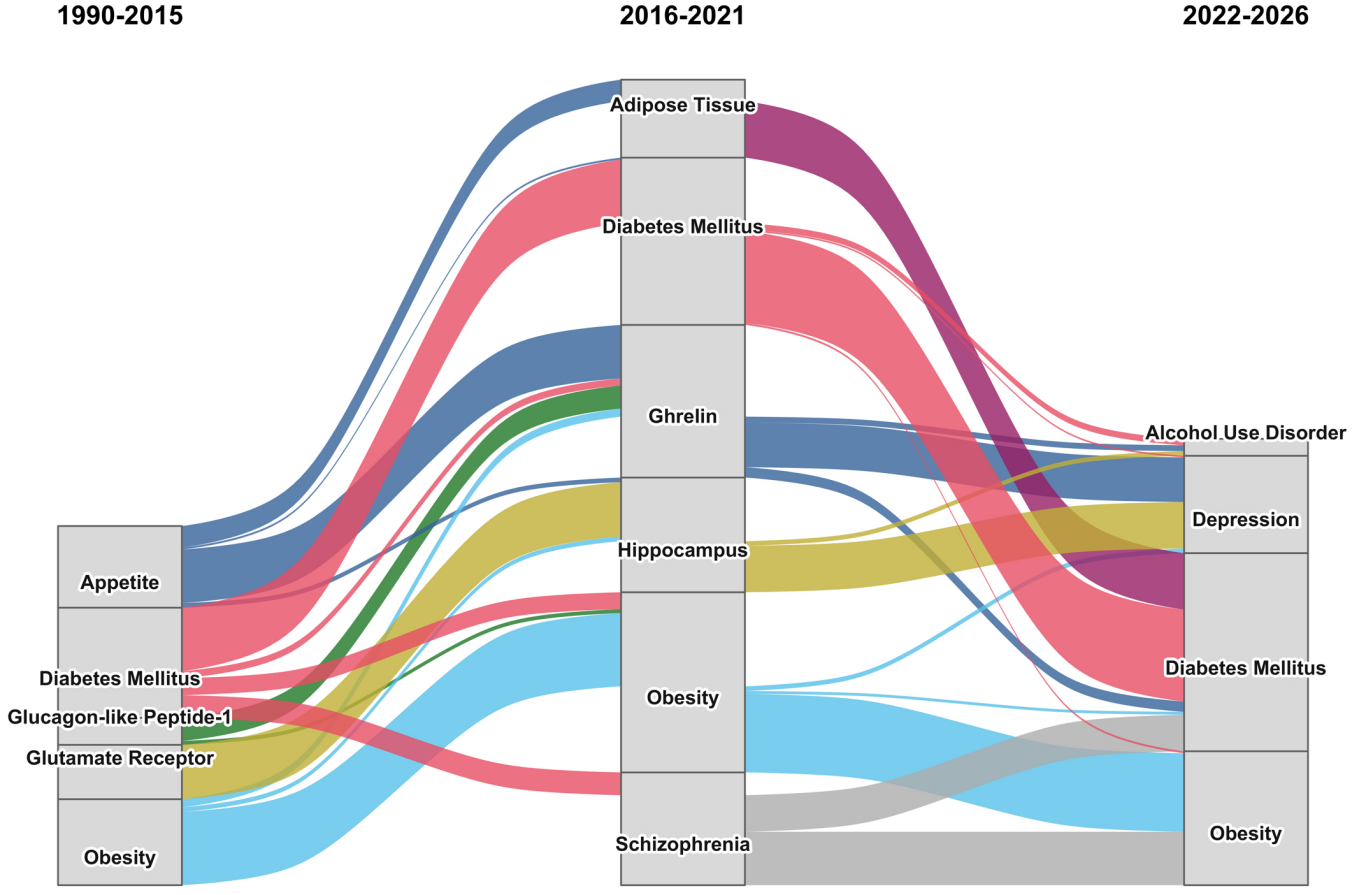
Thematic evolution analysis across three time periods (1990--2015, 2016--2021, 2022--2026). Alluvial diagram showing how research themes merged, split, or emerged over time. Depression emerges as a named cluster in the most recent period (2022--2026), reflecting the integration of psychiatric topics into the field’s dominant research fronts.

Thematic evolution analysis across three time periods revealed a clear trajectory of disciplinary expansion (**Figure 5**). During the first period (1990--2015), the field was organized around clusters including diabetes mellitus, glucagon-like peptide-1, neuroprotection, and anxiety. Psychiatric topics were largely absent from the thematic structure, and the literature was dominated by preclinical studies of GLP-1 receptor biology and early clinical trials for diabetes and weight management. During the second period (2016--2021), the field expanded with clusters including diabetes mellitus, neuroprotection, family medicine, obesity, and a distinct glucagon-like peptide-1 cluster. During the third period (2022--2026), the field reorganized into dominant clusters anchored by diabetes mellitus, obesity, depression, and glucagon-like peptide-1. Depression emerged as a named cluster in the most recent period (2022--2026).

### 3.10 Collaboration networks

The country collaboration map revealed two major collaboration hubs (**Supplementary Figure 2**). The first, anchored by the United States, connected to China, Canada, Australia, Denmark, India, Singapore, and Brazil. The second, centered on the United Kingdom, connected Italy, France, Germany, the Netherlands, Sweden, Spain, and other European countries. The United States served as the primary global collaboration hub.

At the institutional level, prominent collaborative hubs included Harvard University, the National Institutes of Health (NIH), the National Institute on Drug Abuse (NIDA), and Yale University in North America, and the University of Copenhagen, Novo Nordisk, and Karolinska Institutet in Europe (**Supplementary Figure 3**). NIDA was among the most connected institutions. A three-fields plot linked top authors to their keyword and journal associations: McIntyre RS to depression and psychiatry, Jerlhag E to addiction and neuropeptide, and Holst JJ to diabetes and GLP-1 (**Supplementary Figure 4**).

## 4 Discussion

### 4.1 Summary of findings

This bibliometric analysis of 2, 197 documents (1990--2026), drawn from three complementary databases (OpenAlex, Lens.org, PubMed), mapped the intellectual, conceptual, and social structure of research on GLP-1 RAs and psychiatric outcomes. The field has undergone exponential growth, with annual output increasing from fewer than 10 publications before 2004 to nearly 500 in 2025. Thematic mapping revealed that depression has developed internal coherence as a niche theme, while obesity and diabetes mellitus dominate the basic themes quadrant. Thematic evolution demonstrated that depression emerged as a named thematic cluster only in the most recent period (2022--2026).

### 4.2 Growth trajectory and interdisciplinary convergence

The exponential growth pattern observed in this field, particularly the sharp acceleration from 2023 onward, reflects the convergence of several developments. The clinical success and widespread adoption of semaglutide for weight management created a large population of GLP-1 RA users, generating both clinical observations and pharmacoepidemiological data on psychiatric outcomes (2, 7). Concurrently, the maturation of metabolic psychiatry as a discipline provided a theoretical framework for understanding how metabolic interventions might influence psychiatric conditions (26). The distribution of publications across 1, 063 journals spanning endocrinology, psychiatry, neuroscience, pharmacology, and gastroenterology confirms that this is a fundamentally interdisciplinary field, consistent with the core journals identified by Bradford’s Law analysis. This breadth presents both an opportunity and a challenge: the field benefits from diverse perspectives but may be susceptible to fragmentation if interdisciplinary communication is not maintained.

### 4.3 Thematic structure and research frontiers

The positioning of depression within the niche themes quadrant is among the most informative findings of this analysis. In Callon’s centrality-density framework (25), niche themes are internally well-developed but peripheral to the field’s central conversation. This suggests that depression research within the GLP-1 RA literature has established a degree of internal coherence and a recognisable set of core investigators, yet remains somewhat isolated from the dominant metabolic-outcomes research front. This interpretation should be made with caution: position within the strategic diagram reflects the internal density and external centrality of keyword co-occurrence relationships, which index the structural coherence of a research theme rather than its clinical maturity, methodological quality, or the strength of its underlying evidence. By contrast, obesity and diabetes mellitus occupy the basic themes quadrant, serving as central connecting nodes. Appetite occupies the motor themes quadrant, the most mature and central theme, reflecting decades of foundational research on GLP-1 and feeding behaviour.

The shift of GLP-1 RA research focus towards psychiatry and depression may have been augmented by the debate around a potential association between semaglutide and liraglutide and suicidal adverse reactions, which prompted a European Medicines Agency (EMA) review in July 2023 (28). Subsequent analyses of different datasets yielded inconsistent findings, and the EMA ultimately concluded that evidence did not support a causal association between GLP-1 RA treatment and suicidal or self-injurious thoughts and actions (29). The US Food and Drug Administration (FDA) subsequently requested removal of the suicidal behaviour and ideation warning from GLP-1 RA package inserts (30). In parallel, emerging data supported the safety of GLP-1 RAs in patient groups with severe mental illness (31, 32) and broader metabolic utility (33).

Addiction-related keywords (drug, alcohol use disorder) in the co-occurrence network align with the growing evidence base for GLP-1 RAs in substance use disorders (27). Research in this area, led by investigators such as Leggio L and Jerlhag E, bridges preclinical neuroscience and clinical addiction medicine. The prominence of NIDA in the institutional collaboration network supports the interpretation that addiction medicine represents one of the most active research fronts within this field.

### 4.4 Thematic evolution and the emergence of psychiatric research

The thematic evolution trajectory parallels the broader history of GLP-1 RA development. The first period (1990--2015) was dominated by diabetes, GLP-1 biology, and neuroprotection themes, reflecting the drug class’s origins in diabetes treatment and the preclinical characterisation of GLP-1 receptors. The absence of psychiatric themes during this period reflects work being subsumed within neuroscience and pharmacology clusters rather than a complete lack of brain-related research. The emergence of depression as a named thematic cluster in 2022–2026 coincided with large-scale pharmacoepidemiological studies and growing regulatory interest, reflecting a phase of disciplinary convergence in which psychiatric outcomes are studied within the context of cardiometabolic health rather than in isolation.

### 4.5 Comparison with existing bibliometric studies

Five existing bibliometric analyses of GLP-1 RA research (11–15) have focused on cardiovascular outcomes, general semaglutide research, obesity management, and tirzepatide pharmacology; none has examined psychiatric outcomes. The general semaglutide analysis (11) examined 425 documents from Scopus, substantially fewer than the 2, 197 analyzed here, reflecting both our broader scope and the field’s rapid growth since 2023. The dominance of the United States reported in cardiovascular-focused analyses (12, 13) is consistent with our findings. Denmark’s prominence, attributable to Novo Nordisk and Holst JJ’s foundational work on incretin biology, has also been reported in prior GLP-1 RA bibliometric mappings (12). It is also notable that the most highly cited documents in the corpus are predominantly foundational metabolic, pharmacological, and cardiovascular-outcome studies rather than psychiatric-specific work. This reflects both the field’s origins in diabetes and obesity research and the more recent emergence of its psychiatric branch; because citation accrual favours older, metabolically focused papers, raw citation counts are dominated by the broader metabolic literature and, if anything, understate rather than overstate the activity of the younger psychiatric research front.

### 4.6 Strengths

This study has several methodological strengths. To our knowledge, it is the first bibliometric analysis of GLP-1 receptor agonists focused specifically on psychiatric outcomes, filling a notable gap in the existing literature. The multi-database design (OpenAlex, Lens.org, and PubMed/MEDLINE) with parallel title/abstract-restricted Boolean searches strengthens coverage and reproducibility compared with single-database approaches, while remaining entirely open-access. The cleaning pipeline applies rigorous cross-database deduplication, author-name harmonisation, a unified generic-term blacklist, and removal of indexing artifacts, yielding an analytically robust dataset. The analytical framework combines performance analysis, science mapping, and thematic analysis following Donthu et al. (9), and thematic evolution across three time periods provides a dynamic rather than static perspective. All code is version-controlled and will be made publicly available, supporting reproducibility and future longitudinal updates.

### 4.7 Limitations

Several considerations should be kept in mind when interpreting these findings. Cited-reference completeness varies across bibliographic databases and is a known limitation of open-access sources; we therefore focused on performance analysis, science mapping, and thematic analysis rather than co-citation or bibliographic coupling, which require exhaustive reference linking. The three databases use different author-name conventions; we harmonised all sources to a common “SURNAME INITIALS” format with space-separated initials prior to merging, providing consistent author counts, although this does not fully resolve ambiguity for common surname-initial combinations (e.g., “WANG X”). English-language search terms introduce a bias toward English-language literature common to international bibliometric analyses. As with all bibliometric studies, citation counts favour older publications; we reported both mean and median citations and restricted interpretation to relative rankings. Restricting all three searches to title and abstract fields improved cross-database comparability and precision but may have omitted records that address psychiatric outcomes only in their full text or that are retrievable primarily through MeSH indexing, so some relevant work and some thematic representation may be underrepresented. Because OpenAlex does not supply author-assigned keywords, machine-assigned keywords were used as a proxy for that source; although a unified generic-term blacklist and cross-database harmonisation mitigated inconsistency, algorithmically assigned keywords may introduce classification bias and differ in granularity from the author keywords and MeSH descriptors contributed by the other two databases. Finally, thematic map quadrant placement depends on parameter choices; we confirmed the robustness of our cluster structure through sensitivity analysis across minimum frequency thresholds (3, 5, 7), which produced consistent six-cluster solutions, and through a document-type sensitivity analysis restricted to original articles and reviews, which reproduced the descriptive and keyword findings (**Supplementary Tables 4**, **5**).

### 4.8 Implications for research and practice

For researchers, the niche theme status of depression indicates that depression research within the GLP-1 RA field has developed its own methodological identity but remains peripheral to the dominant metabolic research front; bridging this gap, for example, by integrating depression outcomes into large cardiometabolic trials, could shift psychiatric research from niche to motor status. For clinicians, the broad distribution of relevant literature across endocrinology, psychiatry, and neuroscience journals highlights the importance of cross-disciplinary awareness when evaluating GLP-1 RA evidence. For funding agencies, the collaboration network analysis reveals opportunities to strengthen international partnerships bridging North American and European research clusters, and the relatively low international co-authorship rate (14.1%) suggests room for increased cross-border collaboration.

### 4.9 Future research directions

Several research priorities emerge from this analysis. The niche theme status of depression calls for bridging work that integrates psychiatric outcomes with the field’s dominant metabolic research front; large prospective trials with psychiatric co-primary endpoints alongside metabolic endpoints would facilitate this transition. The growing body of addiction-related research would benefit from adequately powered randomised controlled trials of GLP-1 RAs for alcohol, opioid, and other substance use disorders. The thematic map did not identify suicidality as a prominent independent cluster despite its regulatory importance, so focused investigation of GLP-1 RA suicidality signals remains warranted. Longitudinal bibliometric monitoring every two to three years would capture the expected thematic shifts as the field continues to mature.

## 5 Conclusions

This bibliometric analysis of 2, 197 documents published between 1990 and 2026, drawn from three complementary databases, provides the first comprehensive mapping of research on GLP-1 RAs and psychiatric outcomes. The field is undergoing exponential growth and disciplinary convergence. Depression has developed internal coherence as a niche research theme with its own investigators and methodological identity, while obesity and diabetes remain the field’s central basic themes. Bridging these quadrants, moving depression from a well-developed niche toward a more central position, represents a key priority for future research. Large prospective trials with psychiatric co-primary endpoints, expanded clinical evidence for GLP-1 RAs in addiction medicine, and stronger international collaboration across the metabolic and psychiatric research communities will be essential to accelerate this integration.

## Data Availability

The original contributions presented in the study are included in the article/**Supplementary Material**. Further inquiries can be directed to the corresponding author.
