# Supplementary material for: GLP-1 receptor agonists and psychiatric outcomes: a bibliometric analysis of research trends, thematic evolution, and collaboration networks
Source: Front Psychiatry. 2026 Jul 21;17:1857078. doi: 10.3389/fpsyt.2026.1857078 (PMC13433608; doi:10.3389/fpsyt.2026.1857078)
Supplement: Supplementary file 1 [file SupplementaryFile1.docx]

**Supplementary Materials: GLP-1 Receptor Agonists and Psychiatric Outcomes: A Bibliometric Analysis of Research Trends, Thematic Evolution, and Collaboration Networks**

***S0. Database-Specific Search Queries***

All three databases used the same two-block Boolean logic (drug terms AND psychiatric outcome terms), restricted to title and abstract fields to ensure comparability. Database-specific syntax differences are reported below.

**S0.1 OpenAlex.** Searched via the OpenAlex API using the title_and_abstract.search filter. OpenAlex does not support wildcard truncation, so morphological variants were expanded manually. Drug and psychiatric blocks were combined as two separate title_and_abstract.search filters (logical AND):

`

filter=title_and_abstract.search:GLP-1|GLP1|GLP-1RA|glucagon-like peptide-1|glucagon-like peptide 1|semaglutide|liraglutide|dulaglutide|exenatide|tirzepatide|lixisenatide|incretin,title_and_abstract.search:psychiatric|psychiatry|neuropsychiatric|mental health|mental disorder|mental illness|depression|depressive|antidepressant|anxiety|anxious|anxiolytic|suicidal|suicide|suicidality|self-harm|bipolar|mania|manic|psychosis|psychotic|schizophrenia|antipsychotic|mood disorder|affective disorder|obsessive|compulsive|OCD|PTSD|post-traumatic stress|eating disorder|anorexia|anorexic|bulimia|binge eating|addiction|addictive|substance use disorder|alcohol dependence|alcohol use disorder|opioid dependence|opioid use disorder|nicotine dependence|smoking cessation|cocaine use|cocaine dependence|gambling disorder

`

Executed 26 March 2026.

**S0.2 Lens.org.** Run via the Lens.org Scholarly Works search box as a plain Boolean query with wildcard truncation. Because the Lens simple search box automatically searches across title, abstract, keywords, and fields of study, the export was subsequently filtered post-hoc in R to records containing both drug and psychiatric terms in title or abstract only (to match the precision of the other two databases):

`

("GLP-1" OR "GLP1" OR "GLP-1RA" OR "glucagon-like peptide-1" OR "glucagon-like peptide 1" OR semaglutide OR liraglutide OR dulaglutide OR exenatide OR tirzepatide OR lixisenatide OR incretin) AND (psychiatr *OR neuropsychiatr* OR "mental health" OR "mental disorder" OR "mental disorders" OR "mental illness" OR depress *OR antidepressant* OR anxi *OR suicid* OR "self-harm" OR bipolar OR mani *OR psychos* OR psychotic OR schizophren *OR antipsychotic* OR "mood disorder" OR "mood disorders" OR "affective disorder" OR "affective disorders" OR obsessive OR compulsive OR OCD OR PTSD OR "post-traumatic stress" OR "eating disorder" OR "eating disorders" OR anorex *OR bulim* OR "binge eating" OR addict* OR "substance use disorder" OR "substance use disorders" OR "alcohol dependence" OR "alcohol use disorder" OR "opioid dependence" OR "opioid use disorder" OR "nicotine dependence" OR "smoking cessation" OR "cocaine use" OR "cocaine dependence" OR "gambling disorder")

`

Executed 8 April 2026. Year filter 1990–2026; document type filter: Article, Review, Editorial, Letter, Conference Proceedings.

**S0.3 PubMed (MEDLINE).** Run directly in the PubMed search box. The [tiab] field tag restricts to title and abstract only (excluding MeSH-indexed terms) to match the precision of the OpenAlex and Lens searches. Wildcards (*) require ≥ 4 characters before the wildcard in PubMed; short stems were expanded as explicit terms:

`

(("GLP-1"[tiab] OR "GLP1"[tiab] OR "GLP-1RA"[tiab] OR "GLP-1 receptor agonist"[tiab] OR "GLP-1 receptor agonists"[tiab] OR "glucagon-like peptide-1"[tiab] OR "glucagon-like peptide 1"[tiab] OR semaglutide[tiab] OR liraglutide[tiab] OR dulaglutide[tiab] OR exenatide[tiab] OR tirzepatide[tiab] OR lixisenatide[tiab] OR albiglutide[tiab] OR incretin[tiab] OR incretins[tiab]) AND (psychiatr*[tiab] OR neuropsychiatr*[tiab] OR "mental health"[tiab] OR "mental disorder"[tiab] OR "mental disorders"[tiab] OR "mental illness"[tiab] OR depress*[tiab] OR antidepressant*[tiab] OR anxiety[tiab] OR anxious[tiab] OR anxiolytic*[tiab] OR suicid*[tiab] OR "self-harm"[tiab] OR "self harm"[tiab] OR bipolar[tiab] OR mania[tiab] OR manic[tiab] OR psychosis[tiab] OR psychoses[tiab] OR psychotic[tiab] OR schizophren*[tiab] OR antipsychotic*[tiab] OR "mood disorder"[tiab] OR "mood disorders"[tiab] OR "affective disorder"[tiab] OR "affective disorders"[tiab] OR "obsessive-compulsive"[tiab] OR "obsessive compulsive"[tiab] OR OCD[tiab] OR PTSD[tiab] OR "post-traumatic stress"[tiab] OR "posttraumatic stress"[tiab] OR "eating disorder"[tiab] OR "eating disorders"[tiab] OR anorex*[tiab] OR bulim*[tiab] OR "binge eating"[tiab] OR addict*[tiab] OR "substance use disorder"[tiab] OR "substance use disorders"[tiab] OR "alcohol dependence"[tiab] OR "alcohol use disorder"[tiab] OR "opioid dependence"[tiab] OR "opioid use disorder"[tiab] OR "nicotine dependence"[tiab] OR "smoking cessation"[tiab] OR "cocaine use"[tiab] OR "cocaine dependence"[tiab] OR "gambling disorder"[tiab] OR "reward processing"[tiab] OR cognition[tiab] OR "cognitive function"[tiab])) AND ("1990"[Date - Publication] : "2026"[Date - Publication])

`

Executed 8 April 2026. Document type filter applied via sidebar: Journal Article, Review, Editorial, Letter.

**Supplementary Figure S1.** Leading contributors. **(A)** Top 20 most productive sources (journals) by number of documents. **(B)** Top 20 most productive countries by author contributions.

**Supplementary Figure S2.** Country collaboration map. Countries are positioned at their geographic centroids; node size is proportional to each country's total collaboration links, and arc thickness represents the number of co-authored documents between country pairs. The 45 strongest country pairs (minimum two co-authored documents) are shown.

**Supplementary Figure S3.** Institution collaboration network (top 15 institutions). Node size is proportional to collaboration strength; colors represent communities identified by the Louvain algorithm.

**Supplementary Figure S4.** Three-fields plot (alluvial diagram) showing relationships among the top 10 authors (left), top 10 keywords (center), and top 10 journals (right). The width of connecting bands is proportional to the strength of the association.

**Table S1.** Top 10 Most Productive Sources

| **Rank** | **Journal** | **Documents** |
| --- | --- | --- |
| 1 | Journal of the Endocrine Society | 60 |
| 2 | Diabetes | 47 |
| 3 | The American Journal of Gastroenterology | 38 |
| 4 | Gastroenterology | 31 |
| 5 | Frontiers in Endocrinology | 29 |
| 6 | Diabetes Obesity and Metabolism | 28 |
| 7 | Nutrients | 23 |
| 8 | European Neuropsychopharmacology | 20 |
| 9 | International Journal of Molecular Sciences | 19 |
| 10 | The FASEB Journal | 19 |

**Table S2.** Top 10 Most Productive Countries

| **Rank** | **Country** | **Author Contributions** |
| --- | --- | --- |
| 1 | United States | 2,084 |
| 2 | China | 598 |
| 3 | United Kingdom | 399 |
| 4 | Italy | 362 |
| 5 | Denmark | 277 |
| 6 | Canada | 241 |
| 7 | Australia | 229 |
| 8 | Japan | 187 |
| 9 | France | 152 |
| 10 | India | 147 |

**Table S3.** Top 10 Most Productive Authors (post-harmonization)

| **Rank** | **Author** | **Documents** | **Primary Research Focus** |
| --- | --- | --- | --- |
| 1 | McIntyre R S | 16 | Mood disorders, metabolic psychiatry |
| 2 | Fink-Jensen A | 14 | Antipsychotic-related metabolic effects |
| 3 | Jerlhag E | 14 | GLP-1 and addiction/reward pathways |
| 4 | Leggio L | 12 | GLP-1 for alcohol use disorder |
| 5 | Correll C U | 11 | Psychopharmacology, metabolic effects |
| 6 | Lengsfeld S | 11 | GLP-1 clinical trials |
| 7 | Zhang Y | 13 | (common surname; may reflect multiple authors) |
| 8 | Wang Y | 12 | (common surname; may reflect multiple authors) |
| 9 | Wang X | 14 | (common surname; may reflect multiple authors) |
| 10 | Li Y | 11 | (common surname; may reflect multiple authors) |

*Note:* Author counts reflect cross-database harmonization to "SURNAME INITIALS" format. Common East Asian surname-initial combinations may aggregate multiple distinct authors; see Section 4.7 for discussion.

**Table S4.** Document-type sensitivity analysis: full corpus versus original articles and reviews only

| **Metric** | **Full corpus** | **Articles and reviews only** |
| --- | --- | --- |
| Documents | 2,197 | 1,947 |
| Sources (journals) | 1,063 | 987 |
| Authors | 9,269 | 8,667 |
| Timespan | 1990–2026 | 1990–2026 |
| Mean citations per document | 21.9 | 23.9 |
| Field-level h-index | 101 | 100 |

*Note:* Restricting the corpus to original articles and reviews (88.6% of documents) left the field's descriptive structure essentially unchanged.

**Table S5.** Ten most frequent keywords: full corpus versus original articles and reviews only

| **Rank** | **Full corpus (frequency)** | **Articles and reviews only (frequency)** |
| --- | --- | --- |
| 1 | Diabetes mellitus (705) | Diabetes mellitus (634) |
| 2 | Obesity (563) | Type 2 diabetes (496) |
| 3 | Type 2 diabetes (545) | Obesity (492) |
| 4 | Weight loss (281) | Weight loss (255) |
| 5 | Liraglutide (254) | Liraglutide (230) |
| 6 | Insulin (195) | Insulin (176) |
| 7 | Semaglutide (162) | Semaglutide (146) |
| 8 | Insulin resistance (136) | Insulin resistance (119) |
| 9 | Appetite (125) | Appetite (115) |
| 10 | Ghrelin (120) | Ghrelin (109) |

*Note:* All ten keywords are common to both rankings (10/10 overlap); the two lists differ only in the ordering of the second- and third-ranked terms. Frequencies are computed on the merged keyword field before the relevance and generic-term filtering applied to Figure 2B, so absolute counts differ from that figure while the rank structure is directly comparable.
